# Determinants of Mammographic Density Change

**DOI:** 10.1093/jncics/pkz004

**Published:** 2019-02-04

**Authors:** Shadi Azam, Arvid Sjölander, Mikael Eriksson, Marike Gabrielson, Kamila Czene, Per Hall

## Abstract

**Background:**

Mammographic density (MD) is a strong risk factor for breast cancer. We examined how breast cancer risk factors are associated with MD area (cm^2^) change across age.

**Methods:**

We conducted a cohort study of 31 782 Swedish women ages 40–70 years at time of baseline mammogram. Lifestyle and reproductive risk factors were assessed by a web-based questionnaire. MD was measured as dense area using the STRATUS method (mean over the left and right breast). Linear regression analyses with adjustments for age, body mass index (BMI), and menopausal status at baseline were performed to assess the association between breast cancer risk factors and mean baseline MD. To investigate mean MD change across age, linear regression analyses with adjustments for age, BMI, menopausal status, and age at last mammogram were performed. All tests of statistical significance were two-sided.

**Results:**

Except for oral contraceptive use, established lifestyle and reproductive risk factors for breast cancer were associated with baseline mean MD. The overall average annual MD change was −1.0 cm^2^. BMI and physical activity were statistically significantly associated with MD change. Lean women (BMI <20 kg/m^2^) had a mean MD change of −1.13 cm^2^ per year (95% confidence interval = −1.25 to −1.02) compared with −0.46 cm^2^ per year (95% confidence interval = −0.57 to −0.35) for women with BMI 30 or higher. The annual MD change was −0.4 cm^2^ larger in women who were very physically active compared with less physically active women.

**Conclusions:**

Our results indicate that all risk factors for breast cancer, except oral contraceptive use, are associated with baseline MD but that only age, BMI, and physical activity are determinants of MD change.

Mammographic density (MD) reflects the radiologically dense part of a mammogram that consists of epithelial tissue and stroma that appear bright on a mammogram, whereas fat tissue appears dark (1). MD is one of the strongest risk factors of breast cancer (2–7). At a given age and body mass index (BMI), women with very dense breasts (more than 75% density) have a four to six times greater risk of breast cancer compared with women with less dense tissue occupying less than 5% the breast (8,9). MD is a highly heritable trait, but it is also influenced by well-established breast cancer risk factors (10).

Most studies regarding the associations of established risk factors for breast cancer and MD have involved only a single mammographic examination. It has been shown that older age, more children, early pregnancy, postmenopausal status, and elevated BMI are associated with lower MD (11–13). In contrast, high intake of alcohol and use of menopausal hormone therapy (MHT) are associated with greater MD (14,15). MD is a dynamic trait; use of MHT is associated with increased density and use of tamoxifen with decreased density (16–19). In addition, MD decreases with age, a biological process called involution (20,21).

In a longitudinal study by Boyd et al. (11), the average annual reduction in percent density was estimated to be 1%. Few studies have tried to identify determinants of MD change across age (22,23). To study this further, we used the unique prospective Karolinska Mammography Project for Risk Prediction of Breast Cancer (KARMA) cohort, including in total 70 874 women (24), to study the association between established risk factors of breast cancer on both MD and MD change across age.

## Methods

### Study Population

**Figure 1.**
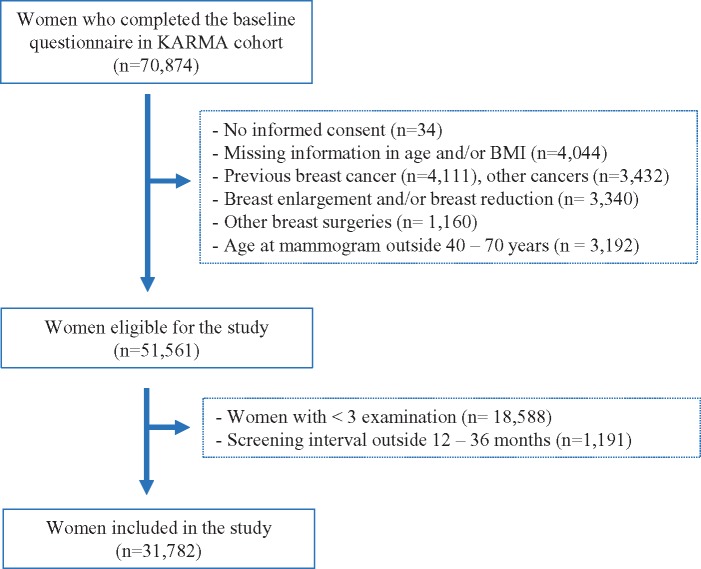
Flow chart describing the exclusion criteria for 70 874 women in the Karolinska Mammography Project for Risk Prediction of Breast Cancer (KARMA) cohort.

The KARMA cohort is a population-based prospective screening cohort initiated in January 2011 and comprises women attending mammography screening or clinical mammography at four hospitals in Sweden (24). Women with a baseline mammogram (n = 70 874) were included in this study. Reasons for exclusion are given in Figure 1. The final analyses included 31 782 Swedish women ages 40–70 years at time of baseline mammogram. The intervals between mammography screening were 12–36 months. All participants signed an informed consent, and the ethical review board at Karolinska Institutet approved the study.

### MD Measurement

We used full-field digital-processed mammograms from the mediolateral oblique view of left and right breasts to measure MD using the area-based STRATUS method (25). We used average STRATUS (over left and right breast) dense area (cm^2^). STRATUS is a fully automated tool developed to analyze digital and analogue images using an algorithm that measures density on all types of images regardless of vendor (25). When studying repeated mammograms from the same individual woman, it is important to consider technical differences between mammograms. As shown in Supplementary Figure 1 (available online), the same amount of breast tissue is not always found in two different mammograms from the same woman. To reduce the influence of this artefact, images should be aligned before density measurements are performed. The concept of alignment is shown in Supplementary Figure 1 (available online) and described in detail elsewhere (25).

### Lifestyle and Reproductive Factors

Participants completed a detailed web-based questionnaire approximately 3 months from date of the baseline mammogram. Lifestyle and reproductive factors were categorizes as: age at baseline (<50, 50–60, >60 years), BMI (<20, 20–24.9, 25–29.9, ≥30 kg/m^2^), smoking status (never, former, current), alcohol consumption (none, 0.1–10, >10 g/d), physical activity (<40, 40–44.9, 45.0–49.9, ≥50 metabolic equivalent of task [MET]-h/d), age at first birth (<20, 20–25, >25 years), number of births (0, 1–2, ≥3), breast-feeding among parous women (0, 1–5, 6–12, >12 months), time since last birth (<10, ≥10 years), menopause status (premenopausal and postmenopausal), age at menarche (<13 or ≥13 years), contraceptive use (yes, no), MHT use (never, former, current), and family history of breast cancer (yes, no). Women reporting no natural menstruation over the past 12 months before the study entry or no menstruation due to oophorectomy were considered postmenopausal. Women with missing information on menstruation status or having no menstruation due to gynecological surgeries other than oophorectomy were considered premenopausal if they were age 50 years or younger and postmenopausal if older than 50 years.

### Statistical Analyses

We used linear regression models to estimate the association of established risk factors for breast cancer with baseline mean MD and 95% confidence intervals. All models were adjusted for age, BMI, and menopausal status at baseline, except the regression model for oral contraceptive use that was additionally adjusted for number of births. For categorical covariates, standard linear regression software produced estimates of the mean in the reference category (the intercept) and the mean difference between each category and the reference. To enhance interpretability, we used the fitted regression models to estimate the mean MD for each risk factor. Technically, this was accomplished with regression standardization (26).

To assess how mean MD changed across age, we fitted local polynomial regression curves using baseline mean MD and age at baseline as the dependent and independent variables, respectively. We fitted one curve for each level of the established breast cancer risk factors listed above. The obtained local polynomial regression curves enables a qualitative (ie, visual) assessment of how mean MD changes as a function of the established breast cancer risk factors, without making strong parametric assumptions. A disadvantage of these curves is that they provide no quantitative measures for testing whether mean MD change differs across levels of established breast cancer risk factors. Another disadvantage is that these curves discard large parts of the data by using only baseline measures of mean dense area.

We carried out a more elaborate density change analysis in two steps. First, one linear regression model was fitted for each woman, regressing all her observed dense area measures during follow-up on her attained ages at these measures. The obtained slopes from these regressions quantify the Woman-specific dense area changes. For instance, a slope equal to −1.5 for a particular woman indicates that the dense area decreased with an average 1.5 cm^2^ per year during follow-up for that woman. Second, the estimated dense area changes (slopes) were regressed on the lifestyle, reproductive, and established breast cancer risk factors listed above, using one linear regression model for each factor. This model was adjusted for age at first and last mammograms, baseline BMI, and baseline menopausal status. Adjustments for age at first and last mammograms are necessary to avoid confounding by age during follow-up, because the age at which the mammograms were taken is strongly related to the estimated dense area change and may also be related to several of the risk factors for breast cancer. Interaction analyses were performed to determine whether determinants of MD change differ by menopausal status.

Lastly, we performed a sensitivity analysis adjusting for both baseline BMI and BMI change among 6427 women for which repeated BMI measures were available. That is, for each woman we have regressed her observed BMI measures during follow-up on her attained ages at these measures. The obtained slopes from these regressions quantify the Woman-specific BMI changes.

All statistical analyses were two-sided and were performed using R version 3.4.1. Statistical significance was measured at significance level 0.05.

## Results

### Baseline Characteristics

Baseline characteristics for the 31 782 women in the study, stratified by menopausal status, are given in Table 1. Because of the large sample size, even small differences were statistically significant. The number of premenopausal and postmenopausal women was nearly the same. In all, 82.2% of participants had completed three rounds of screening and 17.7% had four rounds or more. Approximately 52% of the women had a normal BMI (20–24.9 kg/m^2^), 31.1% were considered overweight, and 11.6% were obese (≥30 kg/m^2^) (Table 1). Almost one-half of the women reported being never smokers and more than one-half reported that they were alcohol drinkers. Compared with premenopausal women (16.0%), postmenopausal women (21.5%) reported that they consumed more than a standard drink (10 g) per day. Only 34.2% of all women were at the lowest level of physical activity (<40 MET-h/d). A larger group of premenopausal women (12.1%) was very physically active (≥50 MET-h) compared with postmenopausal women (8.0%; Table 1). An age at birth of first child older than 25 years was more common among premenopausal (63.1%) women than postmenopausal (43.3%; Table 1) women. However, the proportion of nulliparous women were more or less the same for both groups, 13.4% and 14.0%, respectively. Both premenopausal and postmenopausal women tended to breastfeed longer than 1 year. Postmenopausal women were more likely to have a first-degree relative with a diagnosis of breast cancer (14.4%) than premenopausal women (11.7%).

**Table 1.** Baseline characteristics for all 31 782 women stratified by menopausal status

	No. (%)				
Characteristics	Total	Premenopausal women	Postmenopausal women	P*	*P*†
Women	31 782 (100)	15 932 (50.1)	15 850 (49.8)	<.001	
Screening examinations					
3 rounds	26 134 (82.2)	11 104 (69.6)	15 030 (94.8)	<.001	
≥4 rounds	5648 (17.7)	4828 (30.3)	820 (5.1)	<.001	
Continuous					<.001
Age at baseline, mean (SD), y	53.0 (9.0)	45.6 (4.0)	60.5 (5.9)	<.001	
Age at baseline, range, y	40.0–70.0	40.0-50.0	40.0–70.0		
BMI, mean (SD), kg/m^2^	25.0 (4.0)	24.9 (4.2)	25.2 (3.9)		
BMI, range, kg/m^2^	16.0–54.0	16.70–54.0	16.0–47.9	<.001	
BMI					
<20.0 kg/m^2^	1690 (5.3)	964 (6.0)	726 (4.5)	<.001	
20–24.9 kg/m^2^	16 492 (51.8)	8603 (53.9)	7889 (49.7)	<.001	
25–29.9 kg/m^2^	9893 (31.1)	4515 (28.3)	5378 (33.9)	<.001	
≥30 kg/m^2^	3707 (11.6)	1850 (11.6)	1857 (11.7)	<.001	
Continuous					<.001
Smoking status					
Never	15 656 (49.2)	9025 (56.6)	6631 (41.8)	<.001	
Former	12 277 (38.6)	5111 (32.0)	7166 (45.2)	<.001	
Current	3581 (11.2)	1677 (10.5)	1904 (12.0)	<.001	
Missing	268 (0.8)				
Alcohol consumption, mean (SD), g/d	7.1 (8.5)	7.8 (7.9)	9.5 (9.2)	<.001	
Alcohol consumption, range, g/d	0–142.0	0–142.0	0–134.8		
Alcohol consumption					
0 g/d	5634 (17.7)	2888 (18.1)	2746 (17.3)	<.001	
0.1–10 g/d	19 654 (61.8)	10 279 (64.5)	9375 (59.1)	<.001	
>10 g/d	5964 (18.7)	2555 (16.0)	3409 (21.5)	<.001	
Continuous					<.001
Missing	503 (1.5)				
Physical activity, mean (SD), MET-h/d	42.6 (6.2)	43.1 (6.5)	42.0 (5.9)	<.001	
Physical activity, range, MET-h/d	14.6–97.3	14.7–97.3	14.6–96.1		
Physical activity					
<40 MET-h/d	10 880 (34.2)	5073 (31.8)	5807 (36.3)	<.001	
40.0–44.9 MET-h/d	11 074 (34.8)	5369 (33.6)	5705 (35.9)	<.001	
45.0–49.9 MET-h/d	5741 (18.0)	3174 (19.9)	2567 (16.1)	<.001	
≥50.0 MET-h/d	3203 (10.0)	1935 (12.1)	1268 (8.0)	<.001	
Continuous					<.001
Missing	884 (2.7)				
Age at first birth, mean (SD), y	27.4 (5.2)	28.7 (5.1)	25.9 (4.9)	<.001	
Age at first birth, range, y	14.0–49.0	15 - 49.0	14–48.0		
Age at first birth					
<20.0 y	1378 (4.3)	294 (1.8)	1084 (6.8)	<.001	
20.0–25.0 y	9318 (29.3)	3570 (22.4)	5748 (36.2)	<.001	
>25.0 y	16 934 (53.2)	10 057 (63.1)	6877 (43.3)	<.001	
Continuous					<.001
Missing	4152 (13.0)				
No. of births, mean (SD)	1.9 (1.0)	2.1 (0.7)	2.2 (0.8)	.003	
No. of births, range	0–11	0–10	0–11		
No. of births					
0	3843 (12.0)	2137 (13.4)	2233 (14.0)	<.001	
1–2	19 902 (62.6)	8126 (51.0)	7406 (46.7)	<.001	
≥3	7737 (24.3)	3035 (19.0)	3274 (20.6)	<.001	
Continuous					.003
Missing	300 (0.9)				
Breast-feeding duration, mean (SD),	19.0 (10.0)	20.7 (9.6)	18.1 (9.6)	<.001	
Duration of breast-feeding, range, months	0–87	0–87	0–78		
Duration of breast-feeding‡					
0 mo	523 (1.6)	146 (0.9)	377 (2.3)	.13	
1–5 mo	687 (2.1)	220 (1.3)	467 (2.9)	.04	
6–12 mo	3833 (12.0)	1402 (8.7)	2431 (15.3)	<.001	
>12 mo	20 527 (64.5)	10 682 (67.0)	9845 (62.1)	<.001	
Continuous					<.001
Missing	1862 (5.8)				
Time since last birth, mean (SD), y	21.3 (11.4)	12.8 (6.5)	29.9 (8.4)	<.001	
Time since last birth, range, y	3.0–52.0	3.0–46.0	5.0–52.0		
Time since last birth					
<10 y	5966 (15.9)	4918 (30.8)	148 (0.9)	<.001	
≥10 y	22 788 (71.7)	9077 (56.9)	13 711 (86.5)	<.001	
Continuous					<.001
Missing	3928 (12.3)				
Age at menarche, mean (SD), y	13.0 (1.4)	12.9 (1.4)	13.2 (1.4)	<.001	
Age at menarche, range, y	8.0–20.0	8.0–18.0	8.0–20.0		
Age at menarche					
<13 y	10 849 (34.1)	5989 (37.5)	4860 (30.6)	<.001	
≥13 y	20 094 (63.2)	9568 (60.0)	10 526 (66.4)	<.001	
Continuous					<.001
Missing	839 (2.6)				
Oral contraceptive use					
Never	4045 (12.7)	1304 (8.1)	2741 (17.2)	<.001	
Ever	27 157 (85.4)	14 495 (90.9)	12 662 (79.8)	<.001	
Missing	580 (1.8)				
MTH use					
Never user	24 511 (77.1)	14 650 (91.9)	9861 (62.2)	<.001	
Former user	3998 (12.5)	488 (3.6)	3510 (22.1)	<.001	
Current user	1043 (3.2)	218 (1.3)	825 (5.2)	<.001	
Missing	2230 (7.0)				
Family history of breast cancer					
No	26 799 (84.3)	13 655 (85.7)	13 144 (82.9)	<.001	
Yes	4158 (13.0)	1873 (11.7)	2285 (14.4)	<.001	
Missing	825 (2.5)				

* *P* value for *t* test of means or χ^2^ test of proportions between premenopausal and postmenopausal women; tests were performed at the two-sided .05 significance level. Number of women for each risk factor = The number of women should be added to the number of missing. BMI = body mass index; MET = metabolic equivalent of task; MHT = menopausal hormone therapy; SD = standard deviation.

† *P* value of trend for continuous variables. Tests were performed at the two-sided .05 significance level.

‡ Among parous women.

### Baseline MD

Table 2 shows the association of established risk factors presented in Table 1 with baseline MD. A statistically significantly greater mean baseline MD was seen in younger and leaner women compared with older and obese women (Table 2). Never smokers had a greater MD compared with smokers, and women drinking alcohol had a greater MD compared with nondrinkers.

**Table 2.** Determinants of baseline mammographic dense area in all 31 782 women

Determinants	Women, No. (%)	Mean dense area at baseline in cm^2^ (95% Cl)*	Relative difference in mean dense area, β estimates (95% Cl)*	*P*†	*P*‡
Age baseline, y§					
<50	13 081 (41.1)	32.6 (32.1 to 33.2)	Ref.	Ref.	
50–60	10 053 (31.6)	27.5 (27.1 to 27.9)	−5.14 (−5.86 to −4.41)	<.001	
>60	8648 (27.2)	24.4 (23.8 to 24.9)	−8.25 (−9.19 to −7.31)	<.001	
Continuous					<.001
BMI, kg/m^2^‖					
<20	1690 (5.3)	37.4 (36.4 to 38.4)	Ref.	Ref.	
20.0–24.9	16 492 (51.8)	33.3 (32.9 to 33.6)	−4.09 (−5.15 to −3.04)	<.001	
25.0–29.9	9893 (31.1)	24.5 (24.0 to 24.9)	−12.69 (−13.77 to −11.60)	<.001	
≥30.0	3707 (11.6)	16.2 (15.6 to 16.9)	−20.96 (−22.17 to −19.74)	<.001	
Continuous					<.001
Smoking status					
Never	15 656 (49.2)	28.8 (28.4 to 29.1)	Ref.	Ref.	
Former	12 277 (38.6)	29.0 (28.7 to 29.4)	0.25 (−0.24 to 0.75)	.32	
Current	3581 (11.2)	27.9 (27.2 to 28.5)	−0.94 (−1.70 to −0.17)	.01	
Alcohol consumption, g/d					
0	5634 (17.7)	28.0 (27.5 to 28.6)	Ref.	Ref.	
0.1–10	19 654 (61.8)	28.7 (28.4 to 29.0)	0.65 (0.02 to 1.28)	.04	
>10	5964 (18.7)	29.7 (29.2 to 30.3)	1.67 (0.90 to 2.44)	<.001	
Continuous					<.001
Physical activity, MET-h/d					
<40	10 880 (34.2)	29.3 (28.9 to 29.8)	Ref.	Ref.	
40–44.9	11 074 (34.8)	28.9 (28.5 to 29.3)	−0.47 (−1.02 to 0.08)	.09	
45.0–49.9	5741 (18.0)	28.3 (27.8 to 28.8)	−1.03 (−1.71 to −0.36)	.002	
≥50.0	3203 (10.0)	27.6 (26.8 to 28.3)	−1.75 (−2.58 to −0.92)	<.001	
Continuous					<.001
Age at first birth, y					
<20	1378 (4.3)	26.4 (25.5 to 27.4)	Ref.	Ref.	
20–25	9318 (29.3)	27.6 (27.2 to 28.1)	1.20 (0.03 to 2.37)	.04	
>25	16 934 (53.2)	28.8 (28.5 to 29.2)	2.40 (1.24 to 3.55)	<.001	
Continuous					<.001
No. of children					
0	3843 (12.0)	32.4 (31.7 to 33.2)	Ref.	Ref.	
1–2	19 902 (62.6)	29.0 (28.7 to 29.3)	−3.48 (−4.20 to −2.75)	<.001	
>2	7737 (24.3)	26.5 (26.0 to 27.0)	−5.95 (−6.75 to −5.14)	<.001	
Continuous					<.001
Breast-feeding duration, mo					
0	523 (1.6)	25.6 (23.9 to 27.3)	Ref.	Ref.	
1–5	687 (2.1)	25.5 (24.0 to 26.9)	−0.11 (−2.43 to 2.20)	.92	
6–12	3833 (12.0)	27.5 (26.8 to 28.1)	1.89 (0.02 to 3.75)	.04	
>12	20 527 (64.5)	27.9 (27.6 to 28.2)	2.29 (0.51 to 4.06)	.01	
Continuous					.06
Time since last birth, y					
<10	5066 (15.9)	29.9 (29.2 to 30.7)	Ref.	Ref.	
≥10	22 788 (71.7)	27.9 (27.6 to 28.2)	−1.99 (−2.74 to −1.24)	<.01	
Continuous					<.001
Age at menarche, y					
<13	10 849 (34.1)	28.2 (27.8 to 28.7)	Ref.	Ref.	
≥13	20 094 (63.2)	29.1 (28.8 to 29.4)	0.83 (0.33 to 1.32)	.001	
Continuous					<.001
Oral contraceptive use¶					
Never	4045 (12.7)	29.3 (28.6 to 29.9)	Ref.	Ref.	
Ever	27 157 (85.4)	28.8 (28.5 to 29.0)	−0.53 (−1.23 to 1.67)	.13	
MHT status					
Never user	24 511 (77.1)	28.8 (28.5 to 29.1)	Ref.	Ref.	
Former user	3998 (12.5)	29.4 (28.7 to 30.0)	0.72 (−0.03 to 1.48)	.06	
Current user	1043 (3.2)	31.8 (30.4 to 33.2)	3.90 (2.58 to 5.21)	<.001	
Family history of breast cancer					
No	26 799 (84.3)	28.6 (28.3 to 28.9)	Ref.	Ref.	
Yes	4158 (13.0)	30.3 (29.6 to 30.9)	1.67 (0.99 to 2.36)	<.001	

* Adjusted models: age, BMI, and menopausal status at baseline. BMI = body mass index; CI = confidence interval; MET = metabolic equivalent of task; MHT = menopausal hormone therapy; Ref. = Reference.

† *P* value is for the relative difference in mean baseline dense area (cm^2^), tests were performed at the two-sided .05 significance level.

‡ *P* value of trend for continuous variables for the relative difference in mean baseline dense area (cm^2^), tests were performed at the two-sided .05 significance level.

§ Not adjusted for age at baseline.

‖ Not adjusted for BMI at baseline.

¶ Adjusted for age, BMI, menopausal status, and number of births at baseline.

Physically active, having the first child early in life, having many children, breast-feeding longer than 6 months, having an early menarche, and having the last child 10 years or more years ago were associated with lower MD at baseline (Table 2). Lastly, women using MHT and women with a first-degree relative with breast cancer had a greater MD than those women not using MHT and without a family history of the disease.

### MD Change

**Figure 2.**
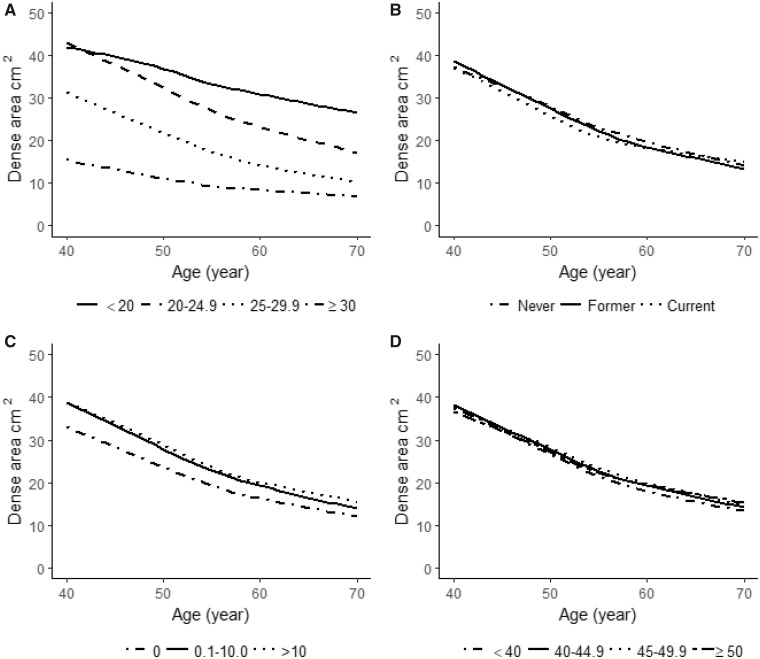
Mean baseline mammographic dense area (cm^2^) as a function of age at mammography screening and established lifestyle factors including: **A**) body mass index ( <20, 20–24.9, 25–29.9, ≥30 kg/m^2^), **B**) smoking status (never, former, current), **C**) alcohol consumption (0, 0.1–10.0, >10 g/d), and **D**) physical activity (<40, 40–44, 45–49.9, ≥50 metabolic equivalent of task-h/d) at study entry.

**Figure 3.**
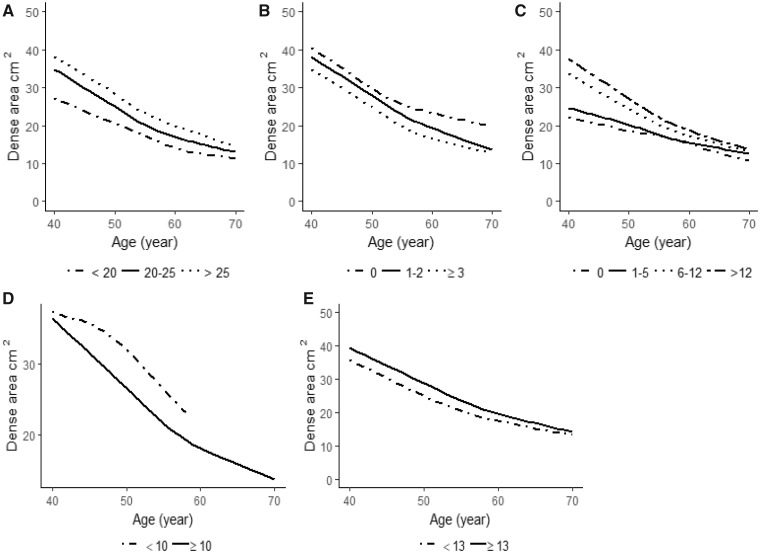
Mean baseline mammographic dense area (cm^2^) as a function of age at mammography screening and reproductive risk factors including: **A**) age at first birth (<20, 20–25, >25 years), **B**) number of children (0, 1–2, ≥3), **C**) breast-feeding duration among parous women (0, 1–5, 6–12, >12 months), **D**) time since last birth (<10, ≥10 years) at study entry, and **E**) age at menarche (<13, ≥13 years).

**Figure 4.**
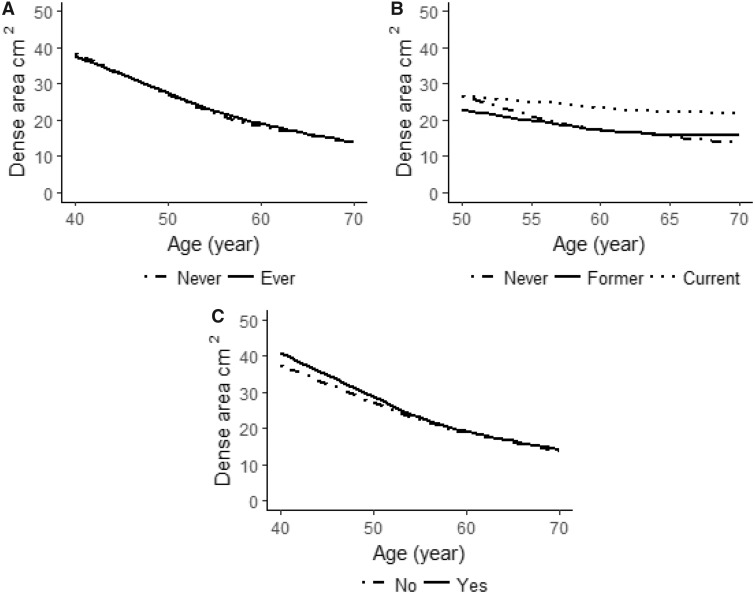
Mean baseline mammographic dense area (cm^2^) as a function of age at mammography screening and **A**) oral contraceptive use (never, ever), **B**) menopausal hormone therapy (MHT) use in postmenopausal women (never, former, current) (because only a few postmenopausal women currently used MHT and were <50 years old [n = 42], we included only postmenopausal women ≥50 years old in this graph), and **C**) family history of breast cancer (yes, no) at study entry.

The overall average annual MD change was –1.0 cm^2^. Table 3 shows the influence of established breast cancer risk factors on MD area change, and Supplementary Tables 1 and 2 (available online) show the same result stratified by menopausal status. The results in Table 3 were adjusted for age, BMI, menopausal status at baseline, and age at last mammogram. In Figures 2, 3, and 4 MD changes are visualized.

**Table 3.** Determinants of mammographic dense area change per year in all 31 782 women

Determinants	Women No. (%)	Mean dense area change in cm^2^/y (95% Cl)*	Relative change of dense area in cm^2^/y, β estimates (95% Cl)*	*P*†	*P*‡
BMI, kg/m^2^§					
<20	1690 (5.3)	−1.13 (−1.25 to −1.02)	Ref.	Ref.	
20.0–24.9	16 492 (51.8)	−1.21 (−1.26 to 1.17)	−0.07 (−0.22 to 0.07)	.30	
25.0–29.9	9893 (31.1)	−0.98 (−1.04 to −0.92)	0.15 (0.00 to 0.31)	.04	
≥30.0	3707 (11.6)	−0.46 (−0.57 to −0.35)	0.67 (0.49 to 0.84)	<.001	
Continuous					<.001
Smoking status					
Never	15 656 (49.2)	−0.98 (−1.04 to −0.94)	Ref.	Ref.	
Former	12 277 (38.6)	−1.09 (−1.15 to −1.04)	−0.10 (−0.18 to −0.03)	.003	
Current	3581 (11.2)	−1.19 (−1.29 to −1.09)	−0.20 (−0.31 to −0.09)	<.001	
Alcohol consumption, g/d					
0	5634 (17.7)	−1.10 (−1.18 to −1.01)	Ref.	Ref.	
0.1–10	19 654 (61.8)	−1.02 (−1.07 to −0.98)	0.07 (−0.01 to 0.16)	.11	
>10	5964 (18.7)	−1.11 (−1.18 to −1.04)	−0.01 (−0.12 to 0.09)	.79	
Continuous					.19
Physical activity, MET-h/d					
<40	10 880 (34.2)	−0.95 (−1.01 to −0.89)	Ref.	Ref.	
40–44.9	11 074 (34.8)	−1.03 (−1.09 to −0.97)	−0.07 (−0.15 to 0.00)	.06	
45.0–49.9	5741 (18.0)	−1.13 (−1.22 to −1.05)	−0.17 (−0.27 to −0.08)	<.001	
≥50	3203 (10.0)	−1.34 (−1.45 to −1.23)	−0.38 (−0.50 to −0.26)	<.001	
Continuous					<.001
Age at first birth, y					
<20	1378 (4.3)	−1.13 (−1.27 to −0.99)	Ref.	Ref.	
20–25	9318 (29.3)	−1.10 (−1.16 to −1.04)	0.03 (−0.13 to 0.20)	.70	
>25	16 934 (53.2)	−1.02 (−1.06 to −0.97)	0.11 (−0.05 to 0.28)	.18	
Continuous					.25
Number of births					
0	3843 (12.0)	−1.11 (−1.21 to −1.00)	Ref.	Ref.	
1–2	19 902 (62.6)	−1.03 (−1.08 to −0.99)	0.07 (−0.03 to 1.17)	.18	
≥3	7737 (24.3)	−1.07 (−1.14 to −1.00)	0.03 (−0.08 to 0.14)	.60	
Continuous					.58
Breast-feeding duration, months					
0	523 (1.6)	−0.82 (−1.04 to −0.60)	Ref.	Ref.	
1–5	687 (2.1)	−0.96 (−1.15 to −0.78)	−0.14 (−0.47 to 0.19)	.40	
6–12	3833 (12.0)	−0.91 (−1.01 to −0.82)	−0.09 (−0.36 to 0.17)	.48	
>12	20 527 (64.5)	−1.05 (−1.09 to −1.01)	−0.22 (−0.48 to 0.02)	.07	
Continuous					.44
Time since last birth, y					
<10	5066 (15.9)	−0.49 (−0.61 to −0.37)	Ref.	Ref.	
≥10	22 788 (71.7)	−1.17 (−1.21 to −1.13)	−0.67 (−0.78 to −0.56)	<.001	
Continuous					.71
Age at menarche, y					
<13	10 849 (34.1)	−1.05 (−1.11 to −0.99)	Ref.	Ref.	
≥13	20 094 (63.2)	−1.05 (−1.10 to −1.01)	−0.00 (−0.00 to 0.00)	.99	
Continuous					.57
Oral contraceptive use					
Never	4045 (12.7)	−0.98 (−1.07 to −0.89)	Ref.	Ref.	
Ever	27 157 (85.4)	−1.06 (−1.10 to −1.03)	−0.08 (−0.18 to 0.01)	.11	
MHT status					
Never user	24 511 (77.7)	−1.08 (−1.12 to −1.04)	Ref.	Ref.	
Former user	3998 (12.5)	−0.95 (1.03 to −0.86)	0.13 (0.02 to 0.24)	.01	
Current user	1043 (3.2)	−1.30 (−1.47 to −1.11)	−0.21 (−0.40 to −0.02)	.02	
Family history of breast cancer					
No	26 799 (84.3)	−1.03 (−1.06 to −0.99)	Ref.	Ref.	
Yes	4158 (13.0)	−1.18 (−1.28 to −1.09)	−0.15 (−0.25 to −0.05)	.001	

* Adjusted model: age, BMI, menopausal status at baseline, and age at the last mammography screening. BMI = body mass index; CI = confidence interval; MET = metabolic equivalent of task; MHT = menopausal hormone therapy, Ref. = Reference.

† *P* value is for the relative dense area change in cm^2^/y; tests were performed at the two-sided .05 significance level.

‡ *P* value of trend for continuous variables for the relative difference in mean baseline dense area (cm^2^); tests were performed at the two-sided .05 significance level.

§ Not adjusted for BMI at baseline.

### Lifestyle Determinants of MD Change

BMI was statistically associated with MD change. Lean women (BMI <20 kg/m^2^) had a mean MD change of –1.13 cm^2^/y (95% CI = –1.25 to –1.02) compared with –0.46 cm^2^/y (95% CI = –0.57 to –0.35) for women with a BMI of 30 or more kg/m^2^ (Table 3). The BMI-dependent difference in MD change is clearly visible in Figure 2.

A borderline statistically significant association of smoking and MD change was seen (Table 3; Supplementary Tables 1 and 2, available online; Figure 2), but point estimates did not differ substantially. Alcohol use did not seem to be associated with MD change (Table 3; Supplementary Tables 1 and 2, available online; Figure 2). In contrast, physically active women had a more pronounced decrease than less active women, particularly among premenopausal women (Table 3; Supplementary Tables 1 and 2, available online; Figure 2). Comparing women with less than 40 MET-h energy expenditure per day to those with 50 or more MET-h/d, the latter group had an annual that was −0.4 cm^2^ larger than the former group (Table 3).

### Reproductive Determinants of MD Change

Reproductive factors such as age at first birth, number of births, breast-feeding, and age at menarche did not seem to be associated with mean MD change (Table 3; Supplementary Tables 1 and 2, available online; Figure 3). Women with 10 or more years since last birth had a statistically significantly greater reduction in yearly MD. This finding did not reach statistical significance when analyzing years since last birth as a continuous variable and when analyzing premenopausal and postmenopausal women separately (Table 3; Supplementary Tables 1 and 2, available online).

### Exogenous Hormones, Family History of Breast Cancer, and MD Change

Use of oral contraceptives or MHT and family history of breast cancer did not seem to have a substantial impact on MD change over time (Table 3; Supplementary Tables 1 and 2, available online; Figure 3). Point estimates did not differ to any greater extent but reached statistical significance in some subgroup analyses.

The results from the interaction analysis show that there was a statistically significant interaction between menopausal status and the following categories: former and current smokers, physically active women, 10 or more years since the last birth, former user of MHT, and finally, women with family history of breast cancer (Supplementary Table 3, available online).

Finally, the results from the sensitivity analyses among the subset of women with repeated measures of BMI show that there is no substantial difference between point estimates when adjusted for both baseline BMI and BMI change (Supplementary Table 4, available online) compared to the results with adjustment for baseline BMI only.

## Discussion

Using a large, well-annotated, prospective cohort, we have shown that a number of established risk factors for breast cancer are associated with MD. At the same time, few of these factors seem to be associated with MD change over time. With the exception of age, only BMI and physical activity had a statistically significant and consistent influence on MD change while controlling for menopause status. Lean and physically active women seemed to decrease more rapidly than obese and sedentary women.

Consistent with other observational studies (23,27–30), we found that a single measure of MD was associated with age and most established risk factors for breast cancer. The exception was oral contraceptive use where we did not see an association with mean baseline MD. Interestingly, we found that longer duration of breast-feeding (>6 months) was associated with greater baseline MD. In line with our results, a Korean cohort study of 122 female twins found that absolute dense area was positively associated with duration of breast-feeding (31). We have previously shown that breast-feeding is associated with greater proportion of epithelial tissue (32). In addition, we found that 10 or more years since last birth was associated with lower baseline MD compared with less than 10 years since last birth. Similar to our result, in a case-control study, Gertig et al. (33) observed a statistically significant increase in proportion of epithelial tissue within approximately 10 years since last birth. In a landmark paper, Lambe et al. (34) found a short-term increased risk of breast cancer after childbirth followed by long-term decrease in risk. This finding could be explained by appearance of epithelial tissues following a pregnancy.

Our results of average annual MD change are in line with a Canadian longitudinal study (11) estimating the average annual reduction in percent density to be 1%. In a nested case-control study, it was shown that age and BMI, unlike other established risk factors for breast cancer, were statistically significantly associated with MD change (23). They showed that overweight and obese women experienced a slower decrease in density over time than women with a BMI less than 25 kg/m^2^ (1.9%, *P *=* *.04 and 3.6%, *P *=* *.01) (23). In another longitudinal cohort study, Kelemen et al. (22) found no statistically significant association between established risk factors for breast cancer and percent MD change, except for age and BMI. They reported that a statistically significant gradual decline in percent MD was observed in younger age and in postmenopausal women with a higher BMI (BMI > 23 kg/m^2^). In agreement with our findings, they also showed that although age at first birth and number of children are associated with baseline percent MD, these factors do not seem to be associated with MD change over time (22).

BMI has been hypothesized to be associated with breast cancer risk through several hormonal-related mechanisms, which may also be relevant to MD change. Increased risk of breast cancer among overweight and obese women may be explained by a higher rate of conversion of androgenic precursors to estrogens through the peripheral aromatization in adipose tissue (35,36). Estrogens are considered to have an important effect on stimulating breast epithelial cell proliferation (37). This is a plausible explanation for a slower age-related decline in MD among obese and overweight women compared with lean women over time. In addition, high levels of insulin and insulin-like growth factors (IGF-I) found among pre- and postmenopausal obese women could stimulate the development and growth of cancer cells (35,38). In a Norwegian cross-sectional study by Bremnes et al. (39), a positive but weak association was shown between mean plasma IGF-I concentration and mean percent and area MD. They observed that women with IGF-I concentrations in the highest quartile had a greater percent of MD compared with women in the lower quartiles (39).

Physical activity is among the few modifiable risk factors for breast cancer. Our result regarding the more pronounced reduction in mammographic dense area among physically active women compared with sedentary women is in contrast with a few longitudinal studies available on physical activity and change in MD (40–42). The results from these studies do not support the hypothesis that physical activity increases the age-related decline in MD. In a longitudinal multiethnic cohort of women (n* *=* *722), Conroy et al. (40) found no association between physical activity and MD change. In a small cohort of postmenopausal Australian women (n* *=* *129), the frequency of participating in exercise for fitness or recreation was not associated with change in percent or area MD (41). Finally, the study conducted on women who participated in the Women’s Health Initiative randomized trial (n* *=* *413) found no association between physical activity and change in percent MD (42). The null findings in these studies may in part be explained by the small sample size, where few women were found in the low and high groups of physical activity. In addition, direct comparisons between studies are challenging due to differences in methods for assessing physical activity. These studies did not align images, as we do using the STRATUS program, before measuring density. Not aligning images might influences the estimates given that physical activity affects the fatty component and thereby the size of the breast.

Several hormonal-related mechanisms behind the association of physical activity and breast cancer have been suggested. Previous findings have shown that physical activity could reduce circulating levels of, and cumulative exposure to, sex steroid hormones during the premenopausal period (43). In addition, physical activity has been shown to decrease estrogen levels among postmenopausal women, in part by reducing the amount of estrogen-producing adipose tissue (44,45). Previous studies found an association between higher levels of circulating estrogen and greater MD in both premenopausal (46) and postmenopausal women (47,48).

Previous studies have shown a positive association between MHT use and increase in MD (17–19). An observational study of 5212 postmenopausal women found that, compared with non-MHT users, women who initiated MHT were more likely to increase MD and women who continuously used MHT were more likely to increase and/or sustain high MD (19). We observed the same pattern in our study with current MHT users sustaining high MD with age (Figure 4). Two randomized studies concluded that a higher MD was seen after estrogen/progestin combination therapy compared with estrogen therapy and never-use of MHT (17,18).

To our knowledge, this is the first large population-based study examining the association between established risk factors for breast cancer and MD change. Strengths of our study are the population-based design, the large number of participants, the detailed information on established breast cancer risk factors, access to repeated and longitudinal measurements of MD from the same women, and measurements of MD after aligning images. The latter feature is most important when analyzing factors that might influence the size of the breast, such as BMI and physical activity. When comparing mammograms from the same woman, it is of outmost importance that images are made comparable before measuring density (Supplementary Figure 1, available online). The same amount of breast tissue is not always seen in different images from the same woman, and an alignment protocol must be used as previously described in detail (25).

There are some limitations in this study that should be considered. The information on established breast cancer risk factors was collected only at the study entry, or at least not more than 3 months before or after entry date, which was the time that the baseline mammogram was taken. Therefore, there is a lack of data on longitudinal change in established risk factors. However, we do not expect dramatic changes in most of the risk factors included in this study, especially not for reproductive risk factors such as age at first birth, number of children, and breast-feeding duration, given the age of the participants. Finally, in this study the information on risk factors for breast cancer was collected using a self-reported questionnaire and therefore is prone to information bias. However, the information bias is most likely nondifferential because women were not aware of their MD measurements or the potential association of these risk factors on MD change. If anything, our estimates could therefore be diluted.

In conclusion, in this large prospective cohort study established risk factors for breast cancer were all associated with MD in the same direction as breast cancer, with the exception of age, BMI in postmenopausal women, and breast-feeding duration, which increased MD, and tobacco, which decreased MD. Physical activity was strongly associated with the baseline MD and MD change over time, as was BMI and age. Collectively, beside age, lean and physically active women have the largest decrease in MD. MD has shown to be a remarkably strong risk factor for breast cancer, whether MD change will be an even better marker of breast cancer risk remains to be studied in large prospective, population-based cohorts.

## Funding

This work was supported by “Märit and Hans Rausing’s Initiative Against Breast Cancer.” The funding agency had no role in the study design, data collection, analyses, and data interoperation, in writing the manuscript, or in the decision to submit the manuscript for publication.

## Notes

Affiliations of authors: Department of Medical Epidemiology and Biostatistics, Karolinska Institutet, Stockholm, Sweden (SA, AS, ME, MG, KC, PH); 

Department of Oncology, Södersjukhuset, Stockholm, Sweden (PH).

The authors declare that they have no conflict of interest.

The authors thank all the participants in the KARMA study and personnel for their devoted work during data collection. They also would like to acknowledge Jose Tapia for helping in data management.

SA participated in the study design, performed the statistical analyses, interpreted the results with support from AS, and drafted the manuscript. AS, ME, MG, KC, and PH participated in the study design and interpretation of the results and helped draft the manuscript. All authors read and approved the final manuscript.

The study was approved by the ethical review board at Karolinska Institutet. Informed consent was obtained from all individual participants included in the study. All experiments comply with the current Swedish laws.

## Supplementary Material

Supplementary Data
