# Supplementary material for: Determinants of Mammographic Density Change
Source: JNCI Cancer Spectr. 2019 Feb 4;3(1):pkz004. doi: 10.1093/jncics/pkz004 (PMC6649843; doi:10.1093/jncics/pkz004)

**Supplementary Table 1.** Determinants of mammographic dense area **change per year** in 15,932 **premenopausal women**

| Determinants | Number of women  No. (%) | Mean dense area change in cm^2^/year (95% Cl)* | Relative change of dense area in cm^2^/year, β estimates (95% Cl)* | P-value † | P-value ‡ |
| --- | --- | --- | --- | --- | --- |
| BMI (kg/m^2^) § |  |  |  |  |  |
| < 20 | 964 (6.0) | -1.59(-1.77 to -1.42) | Ref. | Ref. |  |
| 20.0-24.9 | 8,603 (53.9) | -1.69 (-1.76 to -1.61) | -0.09 (-0.33 to 0.14) | 0.44 |  |
| 25.0-29.9 | 4,515 (28.3) | -1.36 (-1.47 to -1.25) | 0.23 (-0.01 to 0.48) | 0.06 |  |
| ≥30.0 | 1,850 (11.6) | -0.64 (-0.83 to -0.45) | 0.95 (0.67 to 1.23) | <0.001 |  |
| Continuous |  |  |  |  | <0.001 |
| Smoking status |  |  |  |  |  |
| Never | 9,025 (56.6) | -1.41 (-1.48 to -1.33) | Ref. | Ref. |  |
| Former | 5,111 (32.0) | -1.52 (-1.61 to -1.42) | -0.10 (-0.23 to -0.01) | 0.08 |  |
| Current | 1,677 (10.5) | -1.68 (-1.85 to -1.51) | -0.27 (-0.46 to -0.08) | 0.004 |  |
| Alcohol consumption (gram/day) |  |  |  |  |  |
| 0 | 2,888 (18.1) | -1.61 (-1.75 to -1.47) | Ref. | Ref. |  |
| 0.1-10 | 10,279 (64.5) | -1.42 (-1.49 to -1.35) | 0.19 (0.04 to 0.34) | 0.01 |  |
| >10 | 2,555 (16.0) | -1.52 (-1.65 to -1.39) | 0.09 (-0.09 to 0.28) | 0.33 |  |
| Continuous |  |  |  |  | 0.71 |
| Physical activity, (MET-h per day) |  |  |  |  |  |
| < 40 | 5073 (31.8) | -1.31 (-1.41 to -1.21) | Ref. | Ref. |  |
| 40-44.9 | 5369 (33.6) | -1.50 (-1.59 to -1.40) | -0.17 (-0.31 to 0.03) | 0.01 |  |
| 45.0 – 49.9 | 3174 (19.9) | -1.54 (-1.68 to -1.41) | -0.20 (-0.36 to -0.04) | 0.01 |  |
| ≥50 | 1935 (12.1) | -1.70 (-1.85 to -1.54) | -0.38 (-0.56 to -0.19) | <0.001 |  |
| Continuous |  |  |  |  | <0.001 |
| Age at first birth (y) |  |  |  |  |  |
| < 20 | 294 (1.8) | -1.51 (-1.93 to -1.09) | Ref. | Ref. |  |
| 20-25 | 3,570 (22.4) | -1.53 (-1.64 to -1.41) | -0.01 (-0.44 to 0.40) | 0.93 |  |
| > 25 | 10,057 (63.1) | -1.43 (-1.51 to -1.36) | 0.07 (-0.33 to -0.49) | 0.71 |  |
| Continuous |  |  |  |  | 0.97 |
| Number of birth |  |  |  |  |  |
| 0 | 2,137 (13.4) | -1.59 (-1.76 to -1.42) | Ref. | Ref. |  |
| 1-2 | 8,126 (51.0) | -1.45 (-1.52 to -1.38) | 0.13 (-0.04 to 0.31) | 0.13 |  |
| ≥ 3 | 3,035 (19.0) | -1.44 (-1.57 to -1.32) | 0.14 (-0.05 to 0.34) | 0.15 |  |
| Continuous |  |  |  |  | 0.06 |
| Breast feeding duration (m) |  |  |  |  |  |
| 0 | 146 (0.9) | -1.30 (-1.84 to -0.75) | Ref. | Ref. |  |
| 1-5 | 220 (1.3) | -1.74 (-2.15 to -1.32) | -0.43 (-1.17 to 0.30) | 0.24 |  |
| 6-12 | 1,402 (8.7) | -1.46 (-1.66 to -1.26) | -0.16 (-0.76 to 0.44) | 0.60 |  |
| >12 | 10,682 (67.0) | -1.44 (-1.50 to -1.36) | -0.13 (-0.71 to 0.44) | 0.64 |  |
| Continuous |  |  |  |  | 0.49 |
| Time since last birth (y) |  |  |  |  |  |
| <10 | 4,918 (30.8) | -1.44 (-1.56 to -1.32) | Ref. | Ref. |  |
| ≥10 | 9,077 (56.9) | -1.47 (-1.55 to -1.40) | -0.03 (-0.18 to 0.11) | 0.65 |  |
| Continuous |  |  |  |  | 0.15 |
| Age at menarche (y) |  |  |  |  |  |
| <13 | 5,989 (37.5) | -1.51 (-1.61 to -1.42) | Ref. | Ref. |  |
| ≥ 13 | 9,568 (60.0) | -1.44 (-1.51 to -1.36) | 0.07 (-0.04 to 0.19) | 0.21 |  |
| Continuous |  |  |  |  | 0.89 |
| Oral contraceptive use |  |  |  |  |  |
| Never | 1,304 (8.1) | -1.46 (-1.64 to -1.27) | Ref. | Ref |  |
| Ever | 14,495 (90.9) | -1.47 (-1.53 to -1.41) | -0.01 (-0.21 to 0.19) | 0.89 |  |
| MHT Status |  |  |  |  |  |
| Never user | 14,650 (91.9) | -1.47 (-1.53 to -1.41) | Ref. | Ref. |  |
| Former user | 488 (3.6) | -1.53 (-1.85 to -1.20) | -0.05 (-0.37 to 0.27) | 0.74 |  |
| Current user | 218 (1.3) | -1.03 (-1.50 to -0.55) | 0.44 (-0.03 to 0.92) | 0.06 |  |
| Family history of breast cancer |  |  |  |  |  |
| No | 13,655 (85.7) | -1.43 (-1.49 to -1.37) | Ref. | Ref. |  |
| Yes | 1,873 (11.7) | -1.68 (-1.84 to -1.52) | -0.24 (-0.42 to -0.07) | 0.004 |  |

BMI = Body mass index; MET = The metabolic equivalent of task; MHT = Menopausal hormone therapy, CI =

confidence interval, Ref. = Reference, y = Year, m = Months

* Adjusted Model: age at baseline and age at last mammography screening and BMI at baseline

† P-value is for the relative dense area change in cm^2^/year, tests were performed at the two-sided 0.05 significance level

‡ P-value of trend for continuous variables for the relative difference in mean baseline dense area (cm^2^), tests were performed at the two-sided 0.05 significance level

§ Not adjusted for BMI at baseline

**Supplementary Table 2.**  Determinants of mammographic dense area **change per year** in 15,850 **postmenopausal** women

| Determinants | Number of women  No. (%) | Mean dense area change in cm^2^/year (95% Cl)* | Relative change of dense area in cm^2^/year, β estimates (95% Cl)* | P-value † | P-value ‡ |
| --- | --- | --- | --- | --- | --- |
| BMI (kg/m^2^) § |  |  |  |  |  |
| < 20 | 726 (4.5) | -0.83 (-0.97 to -0.68) | Ref. | Ref. |  |
| 20.0-24.9 | 7,889 (49.7) | -0.75 (-0.79 to -0.70) | 0.07 (-0.08 to 0.24) | 0.34 |  |
| 25.0-29.9 | 5,378 (33.9) | -0.56 (-0.62 to -0.50) | 0.26 (0.09 to 0.43) | 0.001 |  |
| ≥30.0 | 1,857 (11.7) | -0.25 (-0.36 to -0.14) | 0.57 (0.38 to 0.76) | <0.001 |  |
| Continuous |  |  |  |  | <0.001 |
| Smoking status |  |  |  |  |  |
| Never | 6,631 (41) | -0.63 (-0.69 to -0.58) | Ref. | Ref. |  |
| Former | 7,166 (45.2) | -0.64 (-0.69 to -0.59) | -0.00 (-0.07 to 0.07) | 0.96 |  |
| Current | 1,904 (12.0) | -0.58 (-0.68 to -0.48) | 0.05 (-0.05 to 0.16) | 0.33 |  |
| Alcohol consumption (gram/day) |  |  |  |  |  |
| 0 | 2,746 (17.3) | -0.61 (-0.69 to -0.53) | Ref. | Ref. |  |
| 0.1-10 | 9,375 (59.1) | -0.63 (-0.68 to -0.59) | -0.01 (-0.11 to 0.07) | 0.70 |  |
| >10 | 3,409 (21.5) | -0.62 (-0.70 to -0.55) | -0.01 (-0.12 to 0.98) | 0.83 |  |
| Continuous |  |  |  |  | 0.43 |
| Physical activity, (MET-h per day) |  |  |  |  |  |
| < 40 | 5,807 (36.3) | -0.61 (-0.67 to -0.55) | Ref. | Ref. |  |
| 40-44.9 | 5,705 (35.9) | -0.61 (-0.66 to -0.55) | 0.00 (-0.07 to 0.08) | 0.93 |  |
| 45.0 – 49.9 | 2,567 (16.1) | -0.66 (-0.74 to -0.57) | -0.04 (-0.14 to 0.05) | 0.38 |  |
| ≥50 | 1,268 (8.0) | -0.79 (-0.92 to -0.67) | -0.18 (-0.31 to -0.04) | 0.007 |  |
| Continuous |  |  |  |  | 0.04 |
| Age at first birth (y) |  |  |  |  |  |
| < 20 | 1,084 (6.8) | -0.76 (-0.88 to -0.63) | Ref. | Ref. |  |
| 20-25 | 5,748 (36.2) | -0.67 (-0.72 to -0.61) | 0.09 (-0.04 to 0.22) | 0.20 |  |
| > 25 | 6,877 (43.3) | -0.58 (-0.63 to -0.52) | 0.17 (0.03 to 0.31) | 0.01 |  |
| Continuous |  |  |  |  | 0.02 |
| Number of birth |  |  |  |  |  |
| 0 | 2,233 (14.0) | -0.57 (-0.67 to -0.46) | Ref. | Ref. |  |
| 1-2 | 7,406 (46.7) | -0.64 (-0.69 to -0.60) | -0.07 (-0.18 to 0.03) | 0.17 |  |
| ≥ 3 | 3,274 (20.6) | -0.62 (-0.69 to -0.56) | -0.05 (-0.17 to 0.06) | 0.35 |  |
| Continuous |  |  |  |  | 0.32 |
| Breast feeding duration (m) |  |  |  |  |  |
| 0 | 377 (2.3) | -0.50 (-0.72 to -0.29) | Ref. | Ref. |  |
| 1-5 | 467 (2.9) | -0.50 (-0.68 to -0.32) | 0.00 (-0.28 to 0.29) | 0.98 |  |
| 6-12 | 2,431 (15.3) | -0.52 (-0.61 to -0.44) | -0.01 (-0.24 to 0.21) | 0.88 |  |
| >12 | 9,845 (62.1) | -0.66 (-0.70 to -0.62) | -0.15 (-0.37 to 0.06) | 0.16 |  |
| Continuous |  |  |  |  | 0.47 |
| Time since last birth (y) |  |  |  |  |  |
| <10 | 148 (0.9) | -0.51 (-1.01 to -0.01) | Ref. | Ref. |  |
| ≥10 | 13,711 (86.5) | -0.63 (-0.67 to -0.59) | -0.12 (-0.48 to 0.23) | 0.50 |  |
| Continuous |  |  |  |  | 0.04 |
| Age at menarche (y) |  |  |  |  |  |
| <13 | 4,860 (30.6) | -0.59 (-0.65 to -0.52) | Ref. | Ref. |  |
| ≥ 13 | 10,526 (66.4) | -0.65 (-0.69 to -0.61) | -0.06 (-0.14 to 0.00) | 0.08 |  |
| Continuous |  |  |  |  | 0.22 |
| Oral contraceptive use |  |  |  |  |  |
| Never | 2,741 (17.2) | -0.60 (-0.68 to -0.51) | Ref. | Ref. |  |
| Ever | 12,662 (79.8) | -0.64 (-0.67 to -0.60) | -0.04 (-0.13 to 0.05) | 0.38 |  |
| MHT Status |  |  |  |  |  |
| Never user | 9,861 (62.2) | -0.61 (-0.65 to -0.56) | Ref. | Ref. |  |
| Former user | 3,510 (22.1) | -0.68 (-0.75 to -0.61) | -0.07 (-0.16 to 0.00) | 0.07 |  |
| Current user | 825 (5.2) | -0.87 (-1.05 to -0.68) | -0.26 (-0.41 to -0.10) | <0.001 |  |
| Family history of breast cancer |  |  |  |  |  |
| No | 13,144 (82.9) | -0.62 (-0.66 to -0.58) | Ref. | Ref. |  |
| Yes | 2,285 (14.4) | -0.66 (-0.70 to -0.57) | -0.03 (-0.13 to 0.05) | 0.43 |  |

BMI = Body mass index; MET = The metabolic equivalent of task; MHT = Menopausal hormone therapy, CI =

confidence interval, Ref. = Reference, y= Year, m = Month

* Adjusted Model: age at baseline and age at last mammography screening and BMI at baseline

† P-value is for the relative dense area change in cm^2^/year, tests were performed at the two-sided 0.05 significance level

** P-value of trend for continuous variables for the relative difference in mean baseline dense area (cm^2^), tests were performed at the two-sided 0.05 significance level

§ Not adjusted for BMI at baseline

**Supplementary Table 3.** Test for interaction between determinants of density change and menopausal status

| Determinants | β estimates of interaction (95% Cl) | P-value * |
| --- | --- | --- |
| BMI (kg/m^2^) |  |  |
| < 20 | Ref. | Ref. |
| 20.0-24.9 | 0.22 (-0.07 to 0.53) | 0.14 |
| 25.0-29.9 | 0.19 (-0.11 to 0.51) | 0.21 |
| ≥30.0 | -0.23 (-0.58 to 0.10) | 0.17 |
| Smoking status |  |  |
| Never | Ref. | Ref. |
| Former | 0.24 (0.09 to 0.38) | <0.001 |
| Current | 0.36 (0.14 to 0.58) | 0.001 |
| Physical activity, (MET-h per day) |  |  |
| < 40 | Ref. | Ref |
| 40-44.9 | 0.19 (0.03 to 0.35) | 0.01 |
| 45.0 – 49.9 | 0.10 (-0.09 to 0.29) | 0.30 |
| ≥50 | 0.04 (-0.19 to 0.29) | 0.70 |
| Time since last birth (y) |  |  |
| <10 | Ref. | Ref. |
| ≥10 | 1.36 (0.86 to 1.86) | <0.001 |
| MHT Status |  |  |
| Never user | Ref. | Ref. |
| Former user | 0.41 (0.12 to 0.71) | 0.005 |
| Current user | -0.18 (-0.64 to 0.27) | 0.44 |
| Family history of breast cancer |  |  |
| No | Ref. | Ref. |
| Yes | 0.30 (0.11 to 0.50) | 0.002 |

BMI = Body mass index; MET = The metabolic equivalent of task; MHT = Menopausal hormone therapy, CI = confidence interval, Ref. = Reference, y = years

* P-value is for the interaction analysis, tests were performed at the two-sided 0.05 significance level

**Supplementary Table 4.**  Determinants of mammographic dense area change per year in 6,427 women

| Determinants | Number of women  No. (%) | Mean dense area change cm2/year (95% Cl)* | Relative change of dense area cm^2^/year, β estimates (95% Cl)* | P-value † | P-value ‡ |
| --- | --- | --- | --- | --- | --- |
| Smoking status |  |  |  |  |  |
| Never | 3,282 (51.0) | -1.10 (-1.20 to -0.99) | Ref. | Ref. |  |
| Former | 2,494 (38.8) | -1.23 (-1.35 to -1.11) | -0.13 (-0.29 to 0.01) | 0.07 |  |
| Current | 625 (9.7) | -1.40 (-1.61 to -1.19) | -0.30 (-0.55 to -0.05) | 0.01 |  |
| Alcohol consumption (gram/day) |  |  |  |  |  |
| 0 | 1,072 (16.6) | -1.11 (-1.32 to -0.90) | Ref. | Ref. |  |
| 0.1-10 | 4,105 (63.8) | -1.22 (-1.30 to -1.12) | -0.10 (-0.30 to 0.09) | 0.29 |  |
| >10 | 1,227 (19.0) | -1.12 (-1.27 to -0.97) | -0.01 (-0.25 to 0.22) | 0.92 |  |
| Continuous |  |  |  |  | 0.57 |
| Physical activity, (MET-h per day) |  |  |  |  |  |
| < 40 | 2,200 (34.2) | -1.11 (-1.24 to -0.98) | Ref. | Ref. |  |
| 40-44.9 | 2,147 (33.4) | -1.16 (-1.28 to -1.04) | -0.04 (-0.21 to 0.12) | 0.60 |  |
| 45.0 – 49.9 | 1,208 (18.7) | -1.23 (-1.39 to -1.06) | -0.11 (-0.31 to 0.09) | 0.28 |  |
| ≥50 | 724 (11.2) | -1.42 (-1.64 to -1.20) | -0.30 (-0.55 to -0.06) | 0.01 |  |
| Continuous |  |  |  |  | 0.01 |
| Age at first birth (y) |  |  |  |  |  |
| < 20 | 179 (2.7) | -1.11 (-1.48 to -0.73) | Ref. | Ref. |  |
| 20-25 | 1,681 (26.1) | -1.30 (-1.45 to -1.16) | -0.19 (-0.63 to 0.24) | 0.37 |  |
| > 25 | 3,684 (57.3) | -1.14 (-1.23 to -1.04) | -0.02 (-0.46 to 0.40) | 0.89 |  |
| Continuous |  |  |  |  | 0.23 |
| Number of birth |  |  |  |  |  |
| 0 | 881 (13.7) | -1.17 (-1.38 to -0.95) | Ref. | Ref. |  |
| 1-2 | 4,038 (62.8) | -1.16 (-1.24 to -1.06) | 0.01 (-0.19 to 0.23) | 0.86 |  |
| ≥ 3 | 1,506 (23.4) | -1.24 (-1.39 to -1.09) | -0.07 (-0.30 to 0.17) | 0.59 |  |
| Continuous |  |  |  |  | 0.32 |
| Breast feeding duration (m) |  |  |  |  |  |
| 0 | 61 (0.94) | -0.33 (-0.97 to 0.31) | Ref. | Ref. |  |
| 1-5 | 115 (1.7) | -0.83 (-1.23 to -0.42) | -0.49 (-1.37 to 0.37) | 0.26 |  |
| 6-12 | 702 (10.9) | -0.95 (-1.15 to -0.74) | -0.62 (-1.35 to 0.11) | 0.09 |  |
| >12 | 4,207 (65.4) | -1.23 (-1.32 to -1.14) | -0.90 (-1.61 to 0.19) | 0.01 |  |
| Continuous |  |  |  |  | 0.01 |
| Time since last birth (y) |  |  |  |  |  |
| <10 | 1,155 (17.9) | -0.73 (-0.94 to -0.53) | Ref. | Ref. |  |
| ≥10 | 4,389 (68.3) | -1.30 (-1.39 to -1.22) | -0.56 (-0.77 to -0.35) | <0.001 |  |
| Continuous |  |  |  |  |  |
| Age at menarche (y) |  |  |  |  |  |
| <13 | 2,271 (35.3) | -1.15 (-1.28 to -1.02) | Ref. | Ref. |  |
| ≥ 13 | 3,979 (61.9) | -1.19 (-1.28 to -1.10) | -0.04 (-0.19 to 0.11) | 0.60 |  |
| Continuous |  |  |  |  | 0.89 |
| Oral contraceptive use |  |  |  |  |  |
| Never | 602 (9.3) | -1.37 (-1.58 to -1.15) | Ref. | Ref. |  |
| Ever | 5,785 (90.0) | -1.16 (-1.24 to -1.09) | 0.20 (-0.04 to 0.44) | 0.10 |  |
| MHT Status |  |  |  |  |  |
| Never user | 5,179 (80.5) | -1.22 (-1.30 to -1.14) | Ref. | Ref. |  |
| Former user | 777 (12.0) | -0.88 (-1.09 to -0.67) | 0.34 (0.09 to 0.58) | 0.006 |  |
| Current user | 222 (3.45) | -1.49 (-1.87 to -1.11) | -0.26 (-0.66 to 0.12) | 0.18 |  |
| Family history of breast cancer |  |  |  |  |  |
| No | 5,487 (85.3) | -1.14 (-1.22 to -1.07) | Ref. | Ref. |  |
| Yes | 798 (12.4) | -1.40 (-1.61 to -1.19) | -0.25 | 0.01 |  |

MET = The metabolic equivalent of task; MHT = Menopausal hormone therapy, CI = confidence interval, Ref. = Reference, y = Year, m = Months

* Adjusted Model: Age, BMI, menopausal status at baseline, BMI change, and age at the last mammography screening

† P-value is for the relative dense area change in cm^2^/year, tests were performed at the two-sided 0.05 significance level

‡ P-value of trend for continuous variables for the relative difference in mean baseline dense area (cm^2^), tests were performed at the two-sided 0.05 significance level

**Supplementary Figure 1.Figure 1** Two mammograms of the same breast were taken 2 minutes apart by the same radiographer. In Frame **A** the mammograms were superimposed to show the difference in breast placement in the mammography machine. In Frame **B**, the two images were digitally aligned to the image showing the smallest breast size (outlined with red in Frame A) prior to density measurement.


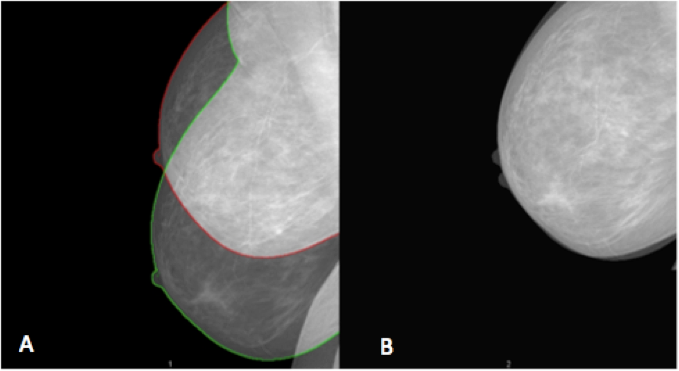

Supplement: Supplementary Data [file pkz004_supp.docx]
